# Correcting for sequence biases in present/absent calls

**DOI:** 10.1186/gb-2007-8-6-r125

**Published:** 2007-06-26

**Authors:** Eugene F Schuster, Eric Blanc, Linda Partridge, Janet M Thornton

**Affiliations:** 1 European Bioinformatics Institute, Wellcome Trust Genome Campus, Hinxton Cambridge CB10 1SD, UK; 2 MRC Centre for Developmental Neurobiology, King's College London, Guy's Hospital Campus, London SE1 1UL, UK; 3 Department of Biology, University College London, Gower Street, London WC1E 6BT, UK

## Abstract

The probe sequence of short oligonucleotides in Affymetrix microarray experiments can have a significant influence on present/absent calls of probesets with absent target transcripts. Probesets enriched for central Ts and depleted of central As in the perfect-match probes tend to be falsely classified as having present transcripts. Correction of non-specific binding for both perfect-match and mismatch probes using probe-sequence models can partially remove the probe-sequence bias and result in better performance of the MAS 5.0 algorithm.

## Abstract 2

Correction of non-specific binding for both PM and MM probes using probe-sequence models can partially remove the probe-sequence bias in Affymetrix microarray experiments and result in better performance of the MAS 5.0 algorithm.

## Background

The Affymetrix GeneChip technology uses a simple method to distinguish 'true' biological signal from background noise. Labeled cRNA transcripts are hybridized to 25 base-pair (bp) oligonucleotide 'probes' covalently bound to the array. Probes are designed in pairs with one probe designed to perfectly match the target transcript (PM probe) and the other designed to measure the non-specific binding signal of its partner PM probe. The mismatch (MM) probe is identical to its partner PM probe except for the central (13th) nucleotide, which is changed to the complementary base. Ideally, the subtraction of MM probe signal from its partner PM probe signal results in the removal of non-specific background and a target transcript specific signal. To gain a more robust target transcript signal, there are typically 11-20 PM-MM probe-pairs within a probeset that query different sequences of the same target transcript. The probeset design also allowed Liu *et al*. [1] to create an algorithm (the MAS 5.0 algorithm) that detects the presence or absence of a target transcript.

PM probe signals that are greater than their partner MM probe signals imply that the target transcripts are present. If the PM signal is equal to its partner MM signal, then it is likely that both PM and MM probes report non-specific binding and the target transcript is absent from the labeled cRNA. For a probe-pair, a discrimination score *R *can be calculated by (PM - MM)/(PM + MM). Liu and colleagues' MAS 5.0 detection algorithm is based on the distribution of *R *scores for every probe-pair within a probeset. The classification of presence or absence is based on *P *values generated with the one-sided Wilcoxon signed rank test [2]. The benefits of the Wilcoxon signed rank test is that it is a non-parametric test, insensitive to outliers and there are well established methods to generate confidence levels (that is, *P *values) [3].

For the present/absent algorithm, the null hypothesis in the Wilcoxon signed rank test is that the distribution of *R *scores for a probeset is symmetrical around τ and that the target transcript is absent from the labeled cRNA. As the median *R *score for a probeset increases above τ, the likelihood that the null hypothesis is true decreases and the chance that the target transcript is present increases. The sensitivity and specificity of the MAS 5.0 algorithm can be adjusted by varying τ. The default value of τ is set to 0.015 based on the medians of discrimination scores for transcripts with concentrations of 0 and 0.25 pM [1]. The setting of τ above zero reduces the number of false positive classifications, as the (PM - MM)/(PM + MM) scores are slightly biased for low *P *values. Liu and colleagues did not pursue the phenomenon further, but recent developments in the understanding of non-specific binding of short oligonucleotides has led us to investigate it.

It is known that up to one-third of MM probes have signal intensities greater than their partner PM probes [4]. While this may contradict the observation that PM probes can be greater than their MM probes when not bound by their target transcripts, it has also led to a likely explanation of the phenomenon. Probe sequence analysis carried out by Naef and Magnasco [5] revealed that in 95% of the cases in which MM probe signal was greater than its partner PM signal, the central nucleotide of the PM probe was a purine (adenine or guanine). Further analysis led them to empirically calculate the contribution of every nucleotide at every position in a 25 nucleotide probe to signal intensity. They found that cytosines (C) contributed to higher signal intensity, especially if they were more central. Conversely, adenines (A) diminished signal intensity, especially if they were more central. Switching a central nucleotide (13th) from A to C could increase the signal intensity by more than two-fold [5]. Given the importance of probe-sequence for signal intensity, we were motivated to determine its importance for present/absent calls and speculated that the present/absent calls would be influenced by probe sequence. One hypothesis is that an empty probeset with many PM probes containing T as the central nucleotide would be falsely called present, as the partner MM probes would have central A nucleotides and, therefore, a lower intensity. Another hypothesis is that probesets with present target transcripts (that is, bound probesets) would be falsely called absent if the PM probes were enriched with central A nucleotides.

In order to carry out a probe-sequence analysis of present/absent calls, we have used a large scale dataset of known composition (the GoldenSpike dataset) [6]. This dataset consists of three replicates of two different cRNA compositions (control and spiked-in) in which all transcripts are known. The cRNA samples are made of 3,859 unique clones of known sequence and were generated from PCR products from the *Drosophila *Gene Collection (DGC; release 1.0) [7]. The absolute concentration of individual cRNA transcripts were not known, but an alignment between the sequences of 3,851 (out of 3859) clones and all PM probe sequences on the Drosgenome1 GeneChip array determined which probesets are called empty and which should be bound by their target transcripts.

**Figure 1:**
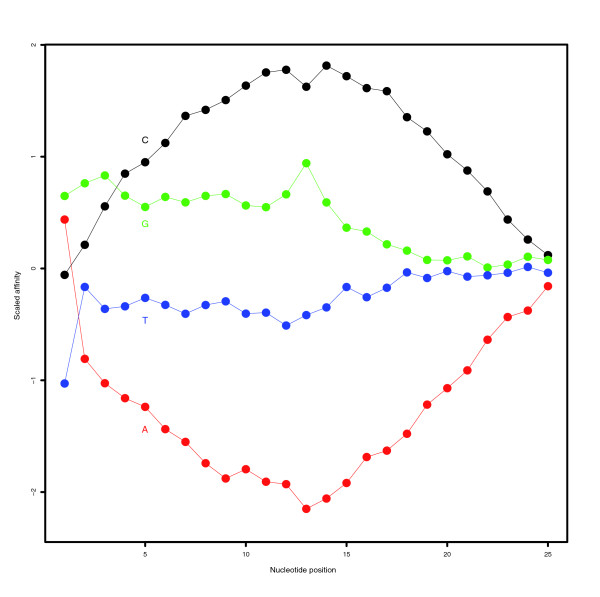
Naef affinities for empty probesets. The probe positions affinities using Naef's model [5] for adenine (red), thymine (blue), cytosine (black) and guanine (green) of empty PM and MM probes in the GoldenSpike dataset. The sum of the affinities as each position is zero, and the affinity of a probe, is based on the sum of the single nucleotide affinities at each position for that probe.

Previous analysis of the GoldenSpike dataset has generated a near complete knowledge of empty and bound probesets, and we were able to classify 10,104 probesets as empty and 3,906 probesets as bound by their target transcript. There were 2,495 probesets that could be aligned to a transcript with equal concentrations in control (C) and spiked-in (S) replicates (fold change = 1 probeset), and there were 1,284 probesets that could be aligned to a transcript with a greater concentration in S replicates (fold change > 1 probeset). There were 127 probesets that could be aligned to multiple transcripts; however, there are also a few probesets that are likely to be falsely classified as empty due to problems in determining the sequence of a small number of clones in the DGC [8]. Additionally, the importance of probe-sequence on signal intensity for empty probesets has been studied and established for the GoldenSpike dataset, and it was found that the Naef probe-sequence model could be used to accurately predict the intensity of non-specific binding of PM and MM probes within empty probesets (Figure 1).

With knowledge of present/absent transcripts and the influence of probe sequence, we could assess the impact of probe sequence on present/absent calls and find methods that would reduce any probe sequence biases. The performance of methods can be assessed by comparing the rate of finding true positives (that is, probesets that can be aligned to transcripts within the GoldenSpike dataset) to the rate of finding false positives (empty probesets) using receiver-operator characteristics (ROC) curves. The use of a large-scale dataset also allows a better assessment of the false discovery rate of the MAS 5.0 present/absent algorithm. Our main goal is to improve the reliability of detecting probesets with absent or present target transcripts.

## Results and discussion

### False positives associated with the MAS 5.0 present/absent algorithm

The MAS 5.0 present/absent calls are determined by the distribution of (PM - MM)/(PM + MM) calculated for each probe-pair within a probeset. Present/absent calls are based on *P *values generated with the one-sided Wilcoxon signed rank test where the null hypothesis is that the distribution of (PM - MM)/(PM + MM) is symmetrical around τ. The mean *P *value of empty probesets is 0.43 when τ is zero and 0.53 when τ is set to the default 0.015 value, confirming the need for a threshold to correct for a slight bias for present calls.

**Figure 2:**
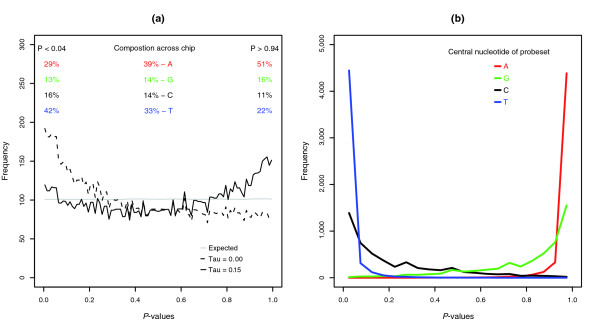
MAS 5.0 present/absent calls. **(a) **The average frequency of *P *values of empty probesets generated by the MAS 5.0 present/absent algorithm when τ = 0.015 (solid black line) and when τ = 0 (dotted line). The average was taken over the six samples. The percentage of central nucleotides in PM probes for empty probesets with *P *values < 0.06, for all empty probesets (similar percentages are present in all probesets), and for empty probesets with *P *values > 0.94 are shown. **(b) ***P *values generated with the Wilcoxon signed rank test for random empty probesets. The PM-MM probe-pairs from empty probesets with fewer than six alignment errors to any transcript in the GoldenSpike dataset were randomly re-assembled into probesets based on the central nucleotide (for example, only central T nucleotides in the PM probes). Symbols and lines are colored according to the central nucleotide.

Probesets classified as having a present or marginally present target transcript have a *P *value less than 0.06 in the MAS 5.0 algorithm, and given a uniform distribution of *P *values for null probesets, 6 out of every 100 probesets with absent target transcripts will be falsely classified as having a present or marginal transcript. The observed distribution of *P *values for empty probesets is clearly not uniformly distributed, and there is a bias for empty probesets to have *P *values near zero or one that results in a slightly higher number of false present calls than expected (Figure 2a). At the 0.06 cutoff, we observed that an average of 7.1 out of every 100 empty probesets were classified as having a present target transcript. However, the difference between expected and observed values may be explained by our lack of complete knowledge of the sequence of every clone in the DGC, as we are lacking the sequence of 8 clones out the 3,859 clones.

Importantly, there is a significant overlap between the empty probesets called present in each sample, and this suggests that false present calls are not random. The total number of empty probesets called absent in all six samples is lower than expected if one assumes the false calls to be random. We observed 1,227 empty probesets with at least one present or marginal transcript call and expected more than 3,600, if the false calls for each sample are random.

### The central nucleotide of PM probes affects present/absent calls of empty probesets

The extreme biases and overlap in false positives are likely to be due to the central nucleotide of the PM probes within a probeset. The signal intensity of 24% of all MM probes is greater than their partner PM probes in all 6 replicates, and 94% of these MM probes are within empty probesets. The central nucleotide of the PM probes in these probe-pairs is a purine (adenine or guanine) 82% of the time.

Across the whole DrosGenome1 GeneChip, 38% of probesets have a central adenosine (A), 14% a central cytosine (C), 14% a central guanine, and 33% a central thymine (T). Empty probesets with *P *values below 0.04 (called present) are enriched for central T nucleotides and depleted of central A nucleotides by almost 10%. Conversely, empty probesets with *P *values greater than 0.96 are enriched for central A nucleotides and depleted of central T nucleotides by more than 10% (Figure 2a).

The bias for high *P *values when the central PM nucleotides of a probeset are pyrimidines (C and T) and the bias for low *P *values when the central PM nucleotides are purines (A and G) can also be demonstrated by creating random empty probesets in which the central PM nucleotide is the same type of nucleotide for all PM probes within the probeset (Figure 2b).

### Present/absent calls of bound probesets

#### False negatives

More than 13% of probesets that can be aligned to fold change = 1 clones (318/2,495) and 3% of probesets that can be aligned to fold change > 1 clones (37/1,284) are falsely classified as having an absent target transcript in all 6 replicate samples. The false negative calls are not random, as there is a greater than 80% overlap between false negative calls in each sample, and more than 75% of the probesets falsely called absent come from the pools added at the lowest concentrations (0.44 μg or less RNA added).

Unexpectedly, the number of fold change (FC) = 1 probesets falsely called absent in S samples is 20% higher (394/323) than in C samples. The majority (85%) of these probesets are also in the PCR-pools in which the amount of RNA added to the final samples was 0.44 μg or less. If the FC = 1 cRNAs were hybridized at the same concentrations in C and S samples, we can only speculate that the differences between C and S samples can account for the higher numbers of false negatives in S samples. The S samples have more total labeled cRNA due to the 'spiked-in' transcripts, and to make the total cRNA concentration the same in each sample, unlabeled poly(C) RNA was added to C samples [6].

#### The influence of the central nucleotide

Assessing the influence of the central nucleotide for probesets bound by their target transcripts is complicated by the fact that the exact concentration of each transcript is not known and the majority of false absent calls are due to the low concentration of the target transcripts. The probesets falsely classified as having absent target transcripts (*P *values > 0.06 in all 6 replicates) are slightly enriched for central A nucleotides (42%) and depleted for central T nucleotides (30%) compared to the whole chip. When considering bound probesets with *P *values > 0.50 in all 6 replicates, 45% of PM probes have a central A and 26% have a central T.

### Alternative present/absent classifications

#### Probeset expression values

**Figure 3:**
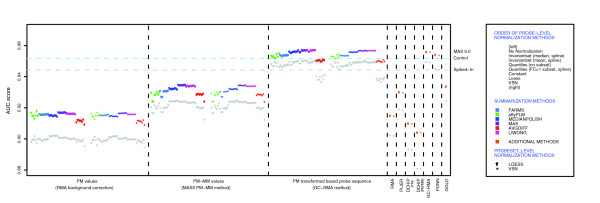
AUC performance for present/absent calls. AUC scores for 301 methods to generate probeset expression values (see Materials and methods and [8] for more information) based on the mean log_2 _value of each probeset for control (C) samples (rainbow colors as in legend). The performance of a method for spiked-in (S) samples (gray) is shown in the same column. True positives are probesets that can be aligned to the DGC clones that were used to create the GoldenSpike dataset. False positives are the remaining empty probesets. The horizontal lines indicate the AUC scores for the mean MAS 5.0 present/absent *P *value for C replicates (blue) and S replicates (gray).

Given that the probe sequence is important for present/absent calls and probe signal intensity, we compared 301 different methods to generate probeset expression values to determine if the expression value cutoff could be used to classify probesets. The majority of methods were based on three different methods for correction of PM values: the robust multichip average (RMA) background correction method [9], in which an estimated background signal is subtracted from all PM probes; the MAS 5.0 method, in which the MM probe intensity is subtracted from its partner PM probe to correct for non-specific binding (NSB); and the GC-RMA method [10], in which PM probe intensities are transformed based on estimates of NSB and probe sequence biases in MM probes. The background/NSB corrections were combined with eight methods for normalization at the probe level, six methods to summarize probe values into probeset values, and loess or variance stabilization normalization [11] at the probeset level (see Materials and methods and [8] for more information). Performance of a method was based on ROC curves, where the rate of finding true positives (bound probesets) is compared to the rate of finding false positives (empty probesets), and performance scores are the area under the ROC curves (AUC). We observed that methods that used probe-sequence based corrections for non-specific binding (GC-RMA [10] and position di-nucleotide nearest neighbor [12] methods) outperformed the other methods and the MAS 5.0 present/absent algorithm. We also observed that the method of background/NSB correction influences performance much more than normalization and summarization methods, and that probeset normalization has very little affect on performance (Figure 3).

**Figure 4:**
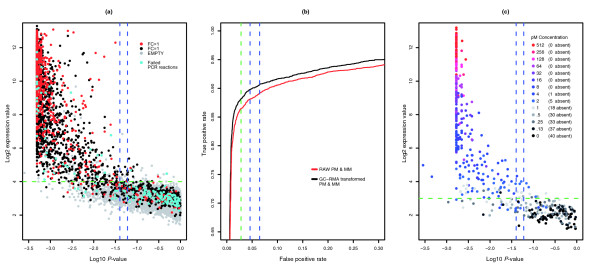
Log_10 _of mean MAS 5.0 present/absent *P *value versus mean log_2 _expression value. **(a) **Plot of log_10 _mean MAS 5.0 present/absent *P *value versus mean log_2 _expression value for control (C) replicates in the GoldenSpike dataset. The default *P *values for absent (0.06) and marginal (0.04) are marked with vertical lines (blue), and suggested expression value cutoffs (99% probesets with *P *values > 0.06 are below the log_2 _cutoff) are marked by a dotted horizontal line (green). **(b) **ROC curves for C replicates using the best performing expression value method (black) and MAS 5.0 *P *values (red). Vertical lines correspond to the false positive rate of cutoff values suggested in (a). **(c) **Plot of log_10 _of mean MAS 5.0 present/absent *P *value versus mean log_2 _expression value for experimental replicates in the Latin-Square dataset. There are 42 spiked-in transcripts that can be aligned to probesets across a range of 14 different concentrations. The number of probesets called absent (MAS 5.0 *P *value < 0.06) is marked in the legend. Lines are as in (a).

The use of expression values as a present/absent classifier is complicated by the need to generate a present/absent cutoff value that will be specific to the GeneChip and the experiment. For example, analysis of the best performing method suggests a log_2 _expression level cutoff value of 4 to remove empty probesets for the GoldenSpike dataset, but the cutoff value for the Latin Square dataset [13] is closer to 3 (Figure 4), possibly due to the content and number of probes in a probeset (14 in Drosgenome1 GeneChip and 16 in HU-133A_tag GeneChip).

The quality of the calls using expression values or MAS 5.0 are also influenced by the content of the samples, as performance is higher on C samples compared to S samples (Figure 3). The unlabeled poly(C) RNA added to the C samples may increase the specificity of the present/absent calls. It is also possible that the higher concentration of labeled RNA in S samples may reduce specificity. In general, the GC-RMA and position di-nucleotide nearest neighbor (PDNN) probe sequence based methods have the smallest differences in performance between C and S samples, and the RMA background correction methods have the largest differences.

#### MAS 5.0 classifications after intensity transformation based on probe sequence

To reduce the influence of the central nucleotide, the intensity of PM and MM probes can be transformed based on Naef's affinities (Figure 1) using the GC-RMA method, and MAS 5.0 present/absent calls can be based on the sequence corrected PM and MM probe intensities. Transformed PM and MM values result in better performance of the MAS 5.0 algorithm, and the distribution of *P *values for empty probesets is similar to the distribution for raw PM and MM values (τ = 0.015) in Figure 2.

**Figure 5:**
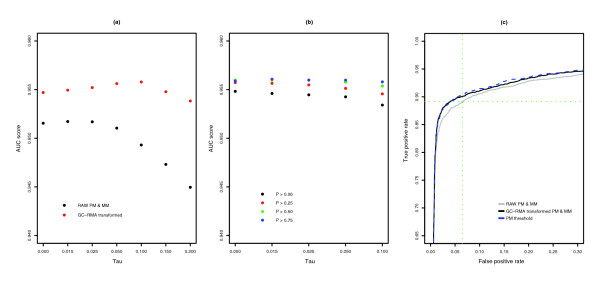
AUC performance of alternative methods. **(a) **AUC scores for the MAS 5.0 present/absent algorithm when raw (black) or GC-RMA transformed (red) PM and MM are used. Scores were generated with a range of τ values. **(b) **AUC scores when MM probe values are replaced by a GC-RMA transformed PM value. The threshold values are based on the mean value of PM probes from probesets that have absent target transcripts. Scores using different stringencies of finding absent transcripts are shown (*P *> 0.06, black; *P *> 0.25, red; *P *> 0.50, green; *P *> 0.75, blue). **(c) **ROC curves based on MAS 5.0 present/absent *P *values when using raw probe values (gray), GC-RMA transformed probe values (black) and threshold probe values (blue dotted line) are shown. Green lines indicate the true (horizontal) and false (vertical) positive rate for the default *P *value cutoff (0.06) to separate 'marginally present' and absent transcripts using raw PM and MM values. AUC and ROC curves are based on the mean *P *value of control replicates.

**Figure 6:**
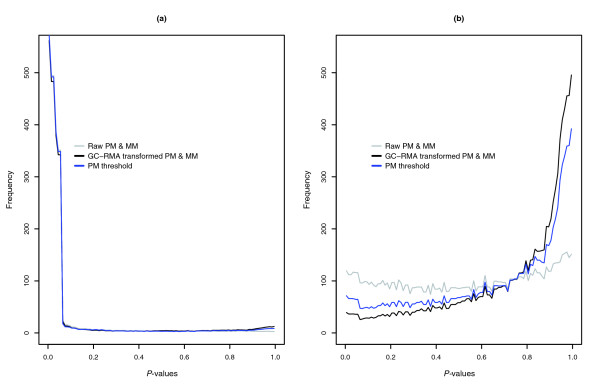
MAS5 present/absent calls for alternative methods. **(a) **The average frequency of *P *values of probesets with present target transcripts generated by the MAS 5.0 present/absent algorithm when using raw PM and MM values (gray), GC-RMA transformed PM and MM values (black) and GC-RMA PM threshold values (blue) are shown. **(b) **The average frequency of *P *values of probesets with absent target transcripts (that is, empty probesets) generated by the MAS 5.0 present/absent algorithm.

For the transformed value, AUC performance peaks when τ is increased from 0.015 to 0.1 (Figure 5a). The change in τ shifts the *P *values of empty probesets towards 1 with little influence on the *P *values of probesets with present transcripts (Figure 6) and increases the ability to discriminate between empty and bound probesets (that is, improve sensitivity and specificity). At the same *P *value or true positive cutoff value, the number of false positives is significantly less when using transformed probe values compared to raw probe values (Figure 5c).

The transformation partially corrects the central nucleotide bias for empty probesets. For the 500 empty probesets with the lowest *P *values (that is, false positives), PM probes have a central T 43% of the time when raw PM and MM values are used and 37% of the time when transformed PM and MM values are used (see Additional data file 1 for a script for performing this transformation).

Given the large number of transcripts that are known to be hybridized in the GoldenSpike dataset, we were able to estimate the false discovery rate of the *P *value cutoffs for present and marginally present transcripts (Table 1). The estimates of false discovery rates are roughly similar in the Latin Square dataset [13] when considering transcripts with concentrations less than 2 pM (data not shown).

**Table 1:** False discovery rates for present and marginally present classifications

	5%			10%			17%		
			
	*P *value	FP	TP	*P *value	FP	TP	*P *value	FP	TP
Raw PM and MM	0.008	1,098	19,398	0.025	2,291	20,182	0.060	4,300	20,687
GC-RMA PM and MM	0.024	1,127	20,013	0.111	2,372	20,607	0.216	4,136	20,944
GC-RMA MM threshold	0.037	1,107	19,969	0.111	2,392	20,621	0.235	4,202	21,003

The number of false positives (FPs) and true positives (TPs) is for a given percentage of false positives out of the total number of positives (5%, 10% and 17% but percentages for each method are not exact due to *P *value granularity) and the corresponding *P *value cutoff for the MAS 5.0 present/absent calls. The calculations are based on a total of 60,624 true negatives (10,104 empty probesets in 6 replicates) and 23,436 true positives (3,906 probesets that can be aligned to transcripts in six replicates). The default *P *value cutoff is 0.060 for separating marginally present from absent transcripts.

#### MAS 5.0 classifications using MM threshold values

To gain a small additional improvement in performance, all MM probe values can be replaced by a threshold value and the MAS 5.0 algorithm can be used to classify target transcripts. The MM threshold value is based on the mean PM value (after GC-RMA transformation) of probesets that are very likely to have absent target transcripts, and the MAS 5.0 present/absent calls are re-calculated with the MM threshold values (Figure 5, Table 1). The MM threshold method also shifts the *P *values of empty probesets towards 1 (Figure 6) and allows a better separation between empty and bound probesets (see Additional data file 1 for a script to perform this method).

## Conclusion

The detection of probesets with absent transcripts is shaped by the central nucleotide of PM probes, with enrichment of central T nucleotides in PM probes resulting in false present calls. This makes sense in light of the way in which PM and MM probes are designed (that is, the central nucleotide of MM probes is complementary to the central nucleotide of PM probes) and the way NSB signal intensity is influenced by probe sequence (that is, T nucleotides contribute to low signal intensity, especially in the middle of the probe, and the complementary A nucleotides contribute to higher signal intensities). The influence of probe-sequence is likely to affect all Affymetrix GeneChip datasets, and we have suggested that transformation of PM and MM probe intensities based on probe-sequence can partially correct for the false positives; the majority of the false positives are associated with PM probes that have a T as the central nucleotide and increase the performance of MAS 5.0 present/absent calls. With the benefit of a large dataset in which hybridized transcripts are known, we can roughly estimate the false discovery rate of the MAS 5.0 present/absent algorithm and suggest more stringent *P *cutoffs that significantly reduce the number of false positives but maintain a similar number of true positives. However, there is still considerable room to improve in our knowledge and handling of short oligonucleotide mRNA expression arrays.

## Materials and methods

### Normalization

R version 2.3.1 [14] and packages within BioConductor [15] were used to generate probeset expression values, except for the PDNN transformation of PM values in Perfect Match [16] and methods available in dChip [17]. For correction of background and/or non-specific binding, we used RMA [9], MAS5 PM-MM [3,18] and GC-RMA [10]. For probe-level normalization, loess, constant [3,18], quantiles [9], variance stabilization normalization (vsn) [11] and invariantset [17] were used. For probe summary into probeset values, medianpolish [9], li-wong [17], tukey-biweight [3,18], farms [19], affyPLM [20], and avgdiff and probeset-level normalization (vsn and loess) were used. In addition, we calculated probeset values with the RMA [9] and GC-RMA [10] methods, the GoldenSpike (GOLD) method [6], which combines the expression values of eight different PM-MM methods, and Probe Logarithmic Intensity Error (PLIER) estimation [21]. All probeset values were imported into R, and normalized using FC = 1 probesets as a subset.

### AUC

AUC scores were generated with ROCR [22]. True positives were defined as probesets that could be aligned to the DGC clones that were part of the GoldenSpike dataset, and true negatives were defined as all other probesets on the DrosGenome1 GeneChip.

### Alternative present/absent methods

Scripts for MAS 5.0 present/absent classifications using transformed PM and MM probe intensities or a PM threshold value are available in Additional data file 1.

## Additional data files

The following additional data are available with the online version of this paper. Additional data file 1 contains scripts for present/absent calls for use in R with BioConductor.

## Supplementary Material

Additional data file 1 Scripts for present/absent calls for use in R with BioConductor. Click here for file
